# Retraction: Early expression of osteopontin glycoprotein on the ocular surface and in tear fluid contributes to ocular surface diseases in type 2 diabetic mice

**DOI:** 10.1371/journal.pone.0357314

**Published:** 2026-09-01

**Authors:** The PLOS One Editors

Following the publication of this article [1], concerns were raised about Fig 6A. Specifically, the following panels appear to originate from the same two images:

- WT Week 4, WT Week 6, OPN-/- Week 4, OPN-/- Week 6, and OPN-/- Week 8
- WT Week 8, WT Week 10, OPN-/- Week 10, and OPN-/- Week 16

The corresponding author stated that an error occurred during the preparation of Fig 6A.

In light of the above unresolved concerns that question the validity and integrity of the published results in [1], the *PLOS One* Editors retract this article.

AD and MN did not agree with the retraction. XYL either did not respond directly or could not be reached.
